# Metagenome quality metrics and taxonomical annotation visualization through the integration of MAGFlow and BIgMAG

**DOI:** 10.12688/f1000research.152290.2

**Published:** 2024-09-23

**Authors:** Jeferyd Yepes-García, Laurent Falquet

**Affiliations:** 1 Swiss Institute of Bioinformatics, Lausanne, Vaud, 1015, Switzerland; 2 Department of Biology, University of Fribourg, Fribourg, Canton of Fribourg, 1700, Switzerland

**Keywords:** Metagenomics, Nextflow, pipeline, dashboard, data analysis.

## Abstract

**Background:**

Building Metagenome–Assembled Genomes (MAGs) from highly complex metagenomics datasets encompasses a series of steps covering from cleaning the sequences, assembling them to finally group them into bins. Along the process, multiple tools aimed to assess the quality and integrity of each MAG are implemented. Nonetheless, even when incorporated within end–to–end pipelines, the outputs of these pieces of software must be visualized and analyzed manually lacking integration in a complete framework.

**Methods:**

We developed a Nextflow pipeline (MAGFlow) for estimating the quality of MAGs through a wide variety of approaches (BUSCO, CheckM2, GUNC and QUAST), as well as for annotating taxonomically the metagenomes using GTDB-Tk2. MAGFlow is coupled to a Python–Dash application (BIgMAG) that displays the concatenated outcomes from the tools included by MAGFlow, highlighting the most important metrics in a single interactive environment along with a comparison/clustering of the input data.

**Results:**

By using MAGFlow/BIgMAG, the user will be able to benchmark the MAGs obtained through different workflows or establish the quality of the MAGs belonging to different samples following *the divide and rule* methodology.

**Conclusions:**

MAGFlow/BIgMAG represents a unique tool that integrates state-of-the-art tools to study different quality metrics and extract visually as much information as possible from a wide range of genome features.

## Introduction

The generation of metagenomics data has increased exponentially within the last ten years, supported by the rapid evolution of next generation sequencing techniques for both short and long reads ( Tremblay et al., 2022). Moreover, to perform the analysis of microbiome data the usual approach involves reconstructing genomes, commonly known as Metagenome–Assembled Genomes (MAGs), from fragmented sequences obtained during DNA extraction. The steps to achieve this goal enclose the cleaning and filtering out of low–quality reads, the assembly of the short sequences into longer and contiguous strands (contigs), plus clustering contigs into bins according to multiple genome–level features such as tetranucleotide frequency, similarity in coverage, GC content, among others ( Yang et al., 2021).

Routinely, the recovered MAGs, independently of the selected workflow to assemble them, should be subject to quality measurements by one or several tools. Contiguity, completeness, and contamination are regular metrics used to classify the MAGs in different categories based on arbitrary criteria ( Haryono et al., 2022). Common thresholds used to separate low (or simply bins), mid or high–quality MAGs are completeness and contamination values generated by tools such as CheckM or CheckM2, *e.g.*, Bowers et al. (2017) established that high–quality MAGs depict levels of contamination below 5% and completeness above 90%. For this manuscript we will use MAGs as a reference to any MAG regardless the category they should be classified in.

Furthermore, given the complexity and abundance of species within some specific environments, *i.e.*, soil or sea sources ( Guseva et al., 2022), more sophisticated tools have been developed to detect the presence of specific marker genes, chimerism and duplication. In addition, proper taxonomical annotation of the MAGs allows to report unique insights related to the composition of the community, contributing to ensure the quality of the assembly and the accuracy of the methods employed to bin the contigs ( Cornet & Baurain, 2022).

Nevertheless, even though many pipelines or methodologies include one or more tools to measure the quality of the MAGs ( Kieser et al., 2020; Krakau et al., 2022; Uritskiy et al., 2018), the visualization and/or analysis of the quality data relies entirely on the user who should be familiar with the type of generated files and how to display the information in a pleasant manner. Additionally, to take advantage of these quality–measuring tools integrated into end-to-end metagenomics pipelines, the users must run the entire workflow, forcing them to carry out the analysis with a specific pipeline. In other cases, the users are required to develop manually their own methodology to accomplish this important step during the metagenome assembly, increasing the risk of lack of reproducibility and the difficulty to test and validate the results. This scenario sets interesting challenges that encases maximizing the scope of the quality assessment, the need of pipelines coupled to visualization tools and boosting reproducibility by wrapping the process with workflow managers ( Wratten et al., 2021).

As a result, we designed a framework to measure the quality of the MAGs generated by different methodologies or belonging to different samples, as well as to visualize the metrics through a web–based interface. This framework carries out the analysis through a Nextflow pipeline (MAGFlow) that takes the MAGs as input to measure their completeness and contamination using machine learning through CheckM2 ( Chklovski et al., 2023), determine the level of single–copy ortholog (SCO) completeness, duplication and fragmentation using BUSCO ( Manni et al., 2021), estimate chimerism and contamination with GUNC ( Orakov et al., 2021), perform a full taxonomical classification by GTDB-Tk2 ( Chaumeil et al., 2022), and produce a full report of the assembly features by QUAST ( Gurevich et al., 2013). After merging the outcomes from these tools, a final. *tsv* file is compiled by MAGFlow, and the user can use it to render an interactive web–based Dash application (BIgMAG).

## Methods

### Implementation

***MAGFlow***

MAGFlow is a pipeline designed to run in any environment, to be portable, easy to install, scalable to the available computational resources and ready to use in local or cloud-based infrastructures. In addition, MAGFlow can be customized through tuning parameters that are available for each tool encompassed in the workflow.

**Figure 1.**
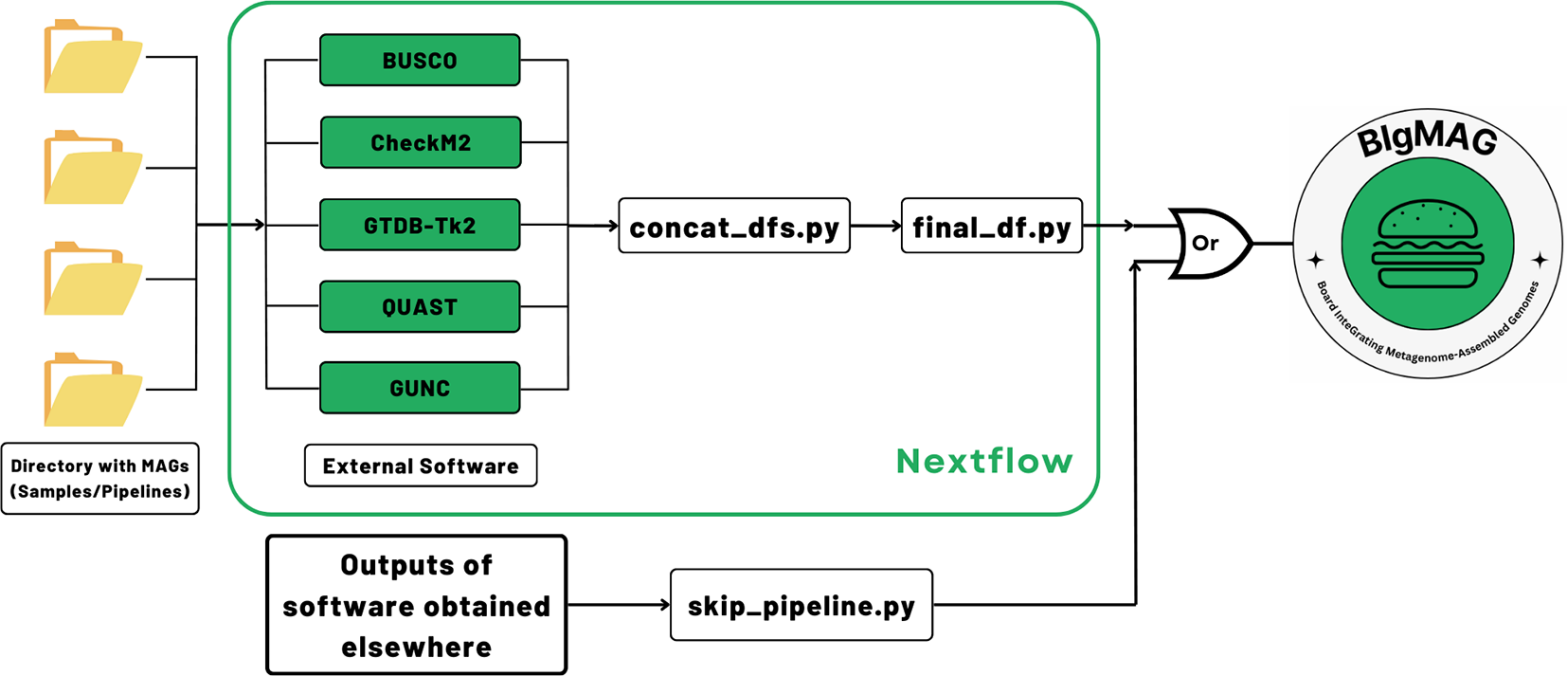
MAGFlow workflow to measure the metagenome quality by different tools, annotate the MAGs taxonomically and render an interactive dashboard using the output from each piece of software.

In order to assess the MAG quality and/or perform the taxonomical annotation, MAGFlow, wrapped by Nextflow (v23.04.0), runs BUSCO (v5.7.0), CheckM2 (v1.0.1), GUNC (v1.0.6), QUAST (v5.2.0) and optionally GTDB-Tk2 (v2.4.0), along with a download of the latest Genome Taxonomy Database ( GTDB, current release: 220), to produce a *.tsv* file that concatenates the outputs from these pieces of software ( Figure 1). The external tools enclosed by MAGFlow are Open Access, and they will remain in such status according to the license provided by their developers.

As input, the pipeline uses genomic files of the MAGs organized in corresponding folders, decompressed ( *.fna*, *.fa*, *.fasta*) or compressed ( *.gz*) obtained from a previous process of assembly/binning. Depending on the type of input, the workflow begins by decompressing the files and checking if there are empty files to be removed from the analysis; the original files will not be modified. Afterwards, the tools mentioned above will be run in parallel according to the allocated resources for each job. The taxonomical annotation is optional for each user given the high demand it represents in terms of memory and storage. Once all the processes are finished, MAGFlow will merge the outcomes from each tool, to end up with a unique *final_df.tsv* file, which is the main input for BIgMAG. Additionally, the pipeline will produce the regular output from each of the tools and HTML execution reports displaying the resource usage, success of each step and timestamps, allowing the user to explore them if required.

In case the user accounts with previously generated BUSCO, CheckM2, GTDB-Tk2, GUNC or QUAST files and their aim is to use them to explore the metrics using BIgMAG, it is possible to achieve this task by just running an additional script ( *skip_pipeline.py*). This script can recognize and process the files into the required *final_df.tsv* by BIgMAG. Directions about how to run MAGFlow or use the *skip_pipeline.py* script are available at https://github.com/jeffe107/MAGFlow.

***BIgMAG***

BIgMAG consists of an interactive Dash application with Plotly as the rendering engine on a regular modern web browser. The layout is divided into 8 sections, 5 of them being plots dedicated exclusively to the tools executed by MAGFlow, and even though BIgMAG can theoretically handle as many samples as the user wishes, it is suggested to include only up to 15 samples per analysis to account with an organized and aesthetic application.

Following the header, that provides a direct link to the software documentation, the dashboard depicts a bar plot summary featuring the percentages of: mid–quality MAGs, high–quality MAGs, MAGs passing GUNC, and if performed GTDB-Tk2, annotated MAGs at the specified taxonomical level and unique annotated MAGs. Moreover, using the raw the numbers (not percentages) of the previously described variables, BIgMAG shows the *p*–value of a Kruskal–Wallis test, a non-parametric statistical test designed to compare two or more groups that allows the study of samples with different size, does not require data with the same underlying distribution and can include several response variables. This *p*–value will be displayed in red when it is lower than the most used threshold for this kind of test, 0.05; however, each user should analyze the significance of this value since in some cases the user may be expecting no difference among the samples, pipelines or binners.

Below the Kruskal–Wallis *p*–value result, a heatmap is presented showing the output of a post–hoc test congruent with the Kruskal–Wallis method to rank the data, the Duncan test, comparing the raw numbers of the same variables analyzed with the Kruskal–Wallis test. It is worthy to mention that the Duncan test is always performed and its results are displayed regardless the *p*–value of the Kruskal–Wallis test. Further, the user may want to expand their data analysis with different statistical methods.

Following the summary section, to present CheckM2 and BUSCO results, scatterplots are used to depict the behavior of genome completeness against contamination levels and complete SCO versus duplicated SCO for CheckM2 and BUSCO, respectively. Moreover, the results from QUAST and GUNC are displayed through boxplots that show the distribution of the data according to a user–selected parameter. The QUAST plot includes the *p*–value of a Welch ANOVA test among samples for the chosen parameter; this type of ANOVA is performed given that if the number of MAGs per sample is uneven, the possibility of violating the homogeneity of variances assumption is increased. Similar to the Kruskal–Wallis *p*–value displayed in the summary section, if the *p*–value is lower than 0.05, it is going to be depicted in red. As discussed previously, the user is in charge of the interpretation of such value depending if they are in the search for significant differences or not among samples, pipelines or binners.

To process the outcomes from GTDB-Tk2, BIgMAG generates a presence/absence matrix representation of annotated taxa at the taxonomical level specified by the user, in a different strategy to extract important data without showing the complete phylogeny of the annotated MAGs. In brief, to create this plot, the summary table generated by GTDB-Tk2 is used to map each taxonomical group annotated by this tool against every sample, displaying, as a result, only if a determined organism at the user-selected rank is present or not in each sample. This approach is valuable in terms of the simplicity it achieves to display this type of information, providing an alternative to the time–consuming branch–collapsing methodology that is usually followed through specialized software packages, *e.g.*, iTOL ( Letunic & Bork, 2021), as shown by Bayer et al. (2020) to condensate the GTDB-Tk2 output.

Following the GTDB-Tk2 plot, a cluster heatmap is displayed, in which the samples are clustered based on their similarity using different parameters provided by BUSCO, CheckM2, GUNC and/or GTDB-Tk2. To render this plot, average values are used, combined with the proportion of MAGs passing the GUNC test and/or the proportion of annotated MAGs by GTDB-Tk2.

At the bottom of the dashboard, the raw data in table format is displayed with the possibility for the user of highlighting cells, deleting rows and filtering the columns with keywords.

Complementary to each plot, there is a series of callbacks that exploits the native interactivity of Plotly and Dash. These callbacks allow filtering out the MAGs according to contamination and completeness threshold levels in the CheckM2 plot, or based on complete SCO and duplicated SCO in the case of the BUSCO figure, selecting the parameter to display the data distribution of each sample in GUNC or QUAST plots and zooming in/out in the GTDB-Tk2 plot by selecting the number of samples to show. Also, it is possible to store all the figures individually directly from the dashboard with the in–built function to download the plots locally. More interactive details will be displayed when the cursor hovers each of the plot zones, i.e., on CheckM2 or BUSCO scatterplots, if GTDB-Tk2 data is found in the *final_df.tsv* file, the taxonomical classification will be also included in the information depicted.

BIgMAG also features the possibility of being compiled as an HTML webpage to be displayed afterwards without requiring any of the processing components installed. However, given that this is not a native feature of Dash, the callbacks cannot be used while the dashboard is stored. Therefore, we developed an additional script ( *app_lite.py*), a lite version of BIgMAG, displaying the same layout as the regular version, although with an additional *Save to html* button below the heading section, and without the components triggering the callbacks. This lite version of BIgMAG can be customized and adjusted by using command line arguments (tutorial available at the repository https://github.com/jeffe107/BIgMAG).

### Operation

MAGFlow/BIgMAG is performed on UNIX-based operating systems, *e.g.*, any Linux distribution or macOS; its operation on Windows 10 or 11 is also possible through Windows Subsystem for Linux ( WSL2). Specifically, MAGFlow demands the installation of Nextflow (≥23.04.0), which in turn requires Java JDK 17 or 19 (recommended version 17.0.3). In addition, it is necessary to account with a software management tool such as Conda (≥23.3.1), Mamba (≥1.3.1) or any container technology including Docker, Singularity, Apptainer, Podman or Charliecloud. After fulfilling these requirements, the user can start the analysis with MAGFlow by providing the directory path where the MAG files are stored or a *.csv* file indicating the different file paths. A terminal example command can be as follows:
nextflow run MAGFlow/main.nf -profile apptainer --files '~/samples/*' --outdir.


Or:
nextflow run MAGFlow/main.nf -profile apptainer –-csv_file '~/samples.csv' --outdir.


The -profile argument must be set according to the software management tool the system user incorporates, and the path to store the output files is completely arbitrary to the user. Details in regard to the required MAG file structure, an example of the *.csv* datasheet, and other configuration options can be found at https://github.com/jeffe107/MAGFlow.

With the aim of providing specific support, MAGFlow has been tested under the system configurations presented in Table 1.

**Table 1.** Different tested configuration settings to perform successful analysis with MAGFlow.

Operating system	Java	Nextflow	Apptainer	Singularity	Docker	Mamba	GTDB-Tk2
Rocky Linux v8.10	OpenJDK 17.0.3	23.10.1	1.3.3-1	–	–	–	Yes
			–	–	–	1.3.1 *	Yes
WSL2 Ubuntu 22.04.4 LTS	OpenJDK 17.0.12	24.04.4	–	3.6.3	–	–	No
			–	–	27.1.1	–	No
			–	–	–	1.5.8 **	No

* Run under Conda 23.3.1 and Python 3.9.15.

** Run under Conda 24.7.1 and Python 3.12.5.

On the other hand, BIgMAG only requires a previous installation of Conda (≥23.3.1), Mamba (≥1.3.1) or pip, plus the availability of any modern browser such as Chrome (≥v124.0.6367.62) or Firefox (≥v124.0.2). Once the user satisfies these requisites, they can install the components and dependencies with the following commands:
pip install -r BIgMAG/requirements.txt


Or:
conda create -n BIgMAG --file BIgMAG/requirements.txt
conda activate BIgMAG


As a result, the user can access the interactive dashboard by providing the path to the *final_df.tsv* file generated by MAGFlow through the following terminal command:
BIgMAG/app.py -p 8050 './final_df.tsv'


The argument -p is included to display BIgMAG on the port of the user preference. The default value is 8050. Once the command is run, the prompt output will indicate the IP direction the user must type on the browser or copy and paste onto it, i.e., http://127.0.0.1:8050/.

## Results

With the aim of demonstrating the usability of MAGFlow/BIgMAG ( v1.0.0), we used it to benchmark the MAG features recovered from a mock community ( ATCC MSA-1003 ^TM^ , Table 2) by 5 different pipelines using only short reads (Illumina), namely Metagenome-ATLAS (ATLAS), DATMA, MetaWRAP, nf–core/mag ( *nf_core_mag_short*) and SnakeMAGs ( Kieser et al., 2020; Benavides et al., 2020; Uritskiy et al., 2018; van Damme et al., 2021; Krakau et al., 2022; Tadrent et al., 2023). Also, the impacts of performing hybrid assembly (Illumina and PacBio) were evaluated by building MAGs from the same mock community with MUFFIN and nf–core/mag ( *nf_core_mag_hybrid*) ( van Damme et al., 2021; Krakau et al., 2022). Additionally, in order to establish the same starting point for all the pipelines processing only short reads, Megahit ( Li et al., 2016) was set as assembly software. In the case of hybrid assembly pipelines, SPAdes ( Nurk et al., 2017) executed the assembly step for both workflows since Megahit does not account with such feature. Further, MetaBAT2 ( Kang et al., 2019) was selected as the binning tool for all the pipelines studied in this section, excluding DATMA since this workflow follows a different strategy to recover the MAGs that groups first the reads using a specific approach called CLAME ( Benavides et al., 2018), to assemble them in batches afterwards.

**Table 2.** Description and details of the samples used to test MAGFlow/BIgMAG.

BioProject	Samples	Origin	Number of reads	Sequencing technology	Reference
PRJNA663614	SRR12687818	Rice soil	10.144.882	Illumina	Fernández-Baca et al., 2021
	SRR12687829		12.652.865		
	SRR12687830		8.286.935		
PRJNA448773	SRR7013867	Wheat soil	15.403.203		Li et al., 2020
	SRR7013874		16.440.929		
PRJNA645385	SRR12192848	Maize rhizosphere	9.483.602		Akinola et al., 2021
	SRR12192849		9.319.178		
	SRR12192850		11.889.426		
PRJNA510527	SRR8359173	Mock community	5.019.157		Portik et al., 2022
	SRR9328980		2.419.037	PacBio	

**Figure 2.**
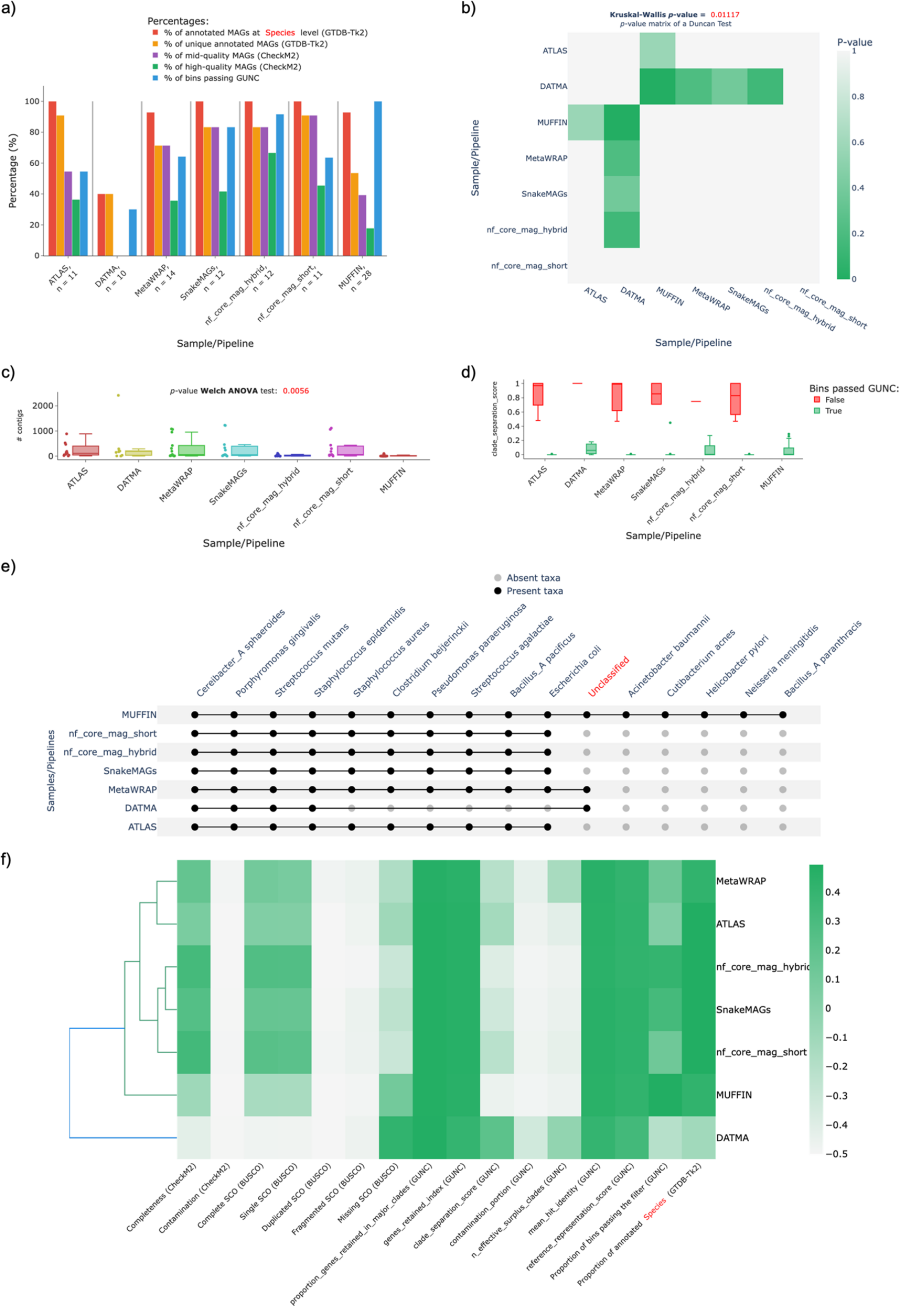
Plots generated by BIgMAG depicting the outputs from different tools used to estimate the quality and annotate taxonomically the MAGs recovered from a sequenced mock community: *a)* bar plot summary of different features ( *see* main text) per pipeline, *b) p*–value matrix of the Duncan test comparing every pipeline against each other, *c)* distribution of the number of contigs per MAG obtained by each pipeline, *d)* data distribution of the CSS per pipeline filtered out by MAGs passing GUNC test, *e)* presence/absence matrix representation depicting annotated taxa in each pipeline at species level and *f)* cluster heatmap of the pipelines in function of their average values for different parameters.

The results obtained by MAGFlow and displayed by BIgMAG for this experiment highlight how pipelines provide similar results notwithstanding they account with different workflow managers. This is the case for MetaWRAP, *nf_core_mag_short*, ATLAS and SnakeMAGs since their quality metrics were similar and the Duncan test did not show significant differences among them ( Figure 2a, b). However, only MUFFIN exhibited significant differences against DATMA considering the parameters the test includes, namely number of: mid–quality or high–quality MAGs (using CheckM2 values), MAGs passing GUNC, annotated MAGs at the specified taxonomical level and unique annotated MAGs (GTDB-Tk2) ( Figure 2b).

Likewise, the *p*–value from the Welch ANOVA performed for the variable *Number of contigs* in the QUAST plot indicates significant differences (taking into account a threshold of 0.005) among the pipelines in regard to this parameter, being probably MUFFIN, DATMA and *nf_core_mag_hybrid* the pipelines that contributed to the significant obtained *p*–value (0.0056) ( Figure 2c).

Another important aspect to consider to compare the pipeline performance is the Clade Separation Score (CSS) calculated by GUNC, a metric that indicates the level of taxonomical chimerism in each contig of a MAG, where MetaWRAP, ATLAS, *nf_core_mag_short* and SnakeMAGs show a more uniform data distribution of the CSS values belonging to the MAGs that passed the GUNC test ( Figure 2d).

Regarding the taxonomical annotation at species level, it is noticeable that DATMA is not as effective to recover the genomes from the mock community as the rest of the workflows, remaining the main proportion of these unannotated ( Figure 2e). Also, MUFFIN outcomes indicate an enhancement in the recovery of less abundant genomes by performing a hybrid assembly with short and long read technologies, since species such as *Acinetobacter baumannii*, *Cutibacterium acnes*, *Helicobacter pylori* and *Neisseria meningitidis* were only detected by this pipeline; this improvement achieved by hybrid assemblies has been proved by Tao et al. (2023) and Liew et al. (2024). Nevertheless, this was not the case for *nf_core_mag_hybrid*, since it did not show a remarkably superior outcome compared to *nf_core_mag_short* in regards of extra species annotated or number of MAGs, although *nf_core_mag_hybrid* provided a ~20% increase in the proportion of high–quality MAGs and a higher percentage ~30% of MAGs passing GUNC test ( Figure 2a).

As a result, the observations afore-mentioned contribute to the pipeline grouping displayed in the cluster heatmap, in which the metrics of the outcomes from MetaWRAP seem to be closer to the values obtained with ATLAS, and more dissimilar from the cluster that encompasses nf–core/mag ( *short* and *hybrid*) and SnakeMAGs; MUFFIN and DATMA represent the most different workflows in regard to the quality features depicted by the MAGs assembled with these pipelines ( Figure 2f).

In terms of performance, MAGFlow ( v1.0.0) takes approximately 18 minutes to analyze the MAGs obtained from the mock community on an HPC cluster with 128 CPUs and 500 GB of available memory using the local executor and the default configuration that can be found on the file *MAGFlow/conf/base.config* at the repository ( https://github.com/jeffe107/MAGFlow). Downloading the GTDB-Tk2 database can take up to 4 hours, while retrieving the GUNC database takes ~1 hour depending on the bandwidth of the user Internet network.

In order to show another example of the utility of MAGFlow/BIgMAG (v1.0.0), we used nf–core/mag to recover MAGs from several public metagenomics datasets generated from different crop rhizospheres or soil including rice, wheat and maize ( Table 2). Additionally, two modes of assembly and binning were considered, namely SASB as single assembly/single binning and CACB as co-assembly/co-binning. Considering the intensive computational demands by the co-assembly process, only Megahit was used as assembly software and MetaBAT2 as the binning tool, allowing the same experimental setting for both SASB and CACB modes. Besides, the raw reads were previously cleaned using fastp ( Chen et al., 2018), and a host removal was performed with bowtie2 ( Langmead & Salzberg, 2012), indexing the proper host genome according to the type of crop the samples were obtained from. The NCBI RefSeq assembly accession numbers of the host genomes used to map the corresponding reads are GCF_018294505.1 (wheat), GCF_000005425.2 (rice) and GCF_902167145.1 (maize).

**Figure 3.**
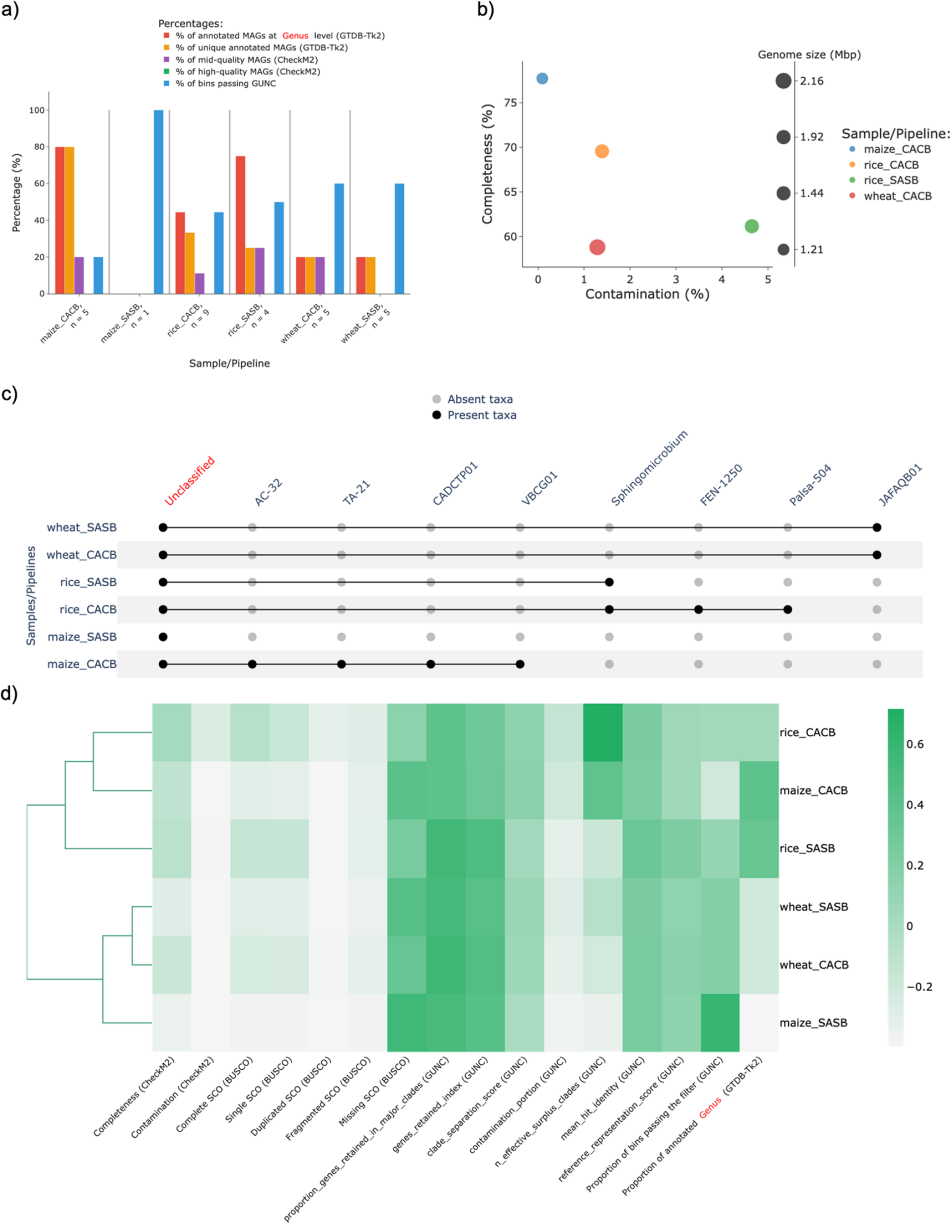
Results of the experiment to explore the recovered MAGs by nf–core/mag from different soil/rhizosphere samples using MAGFlow/BIgMAG: *a)* bar plot summary of different features ( *see* main text) per sample, *b)* scatterplot of the completeness level against contamination portion of mid–quality MAGs, *c)* presence/absence matrix representation depicting annotated taxa in each sample at genus level and *d)* cluster heatmap of the samples in function of their average values for different parameters. The names of the samples are represented by merging the origin of the sample (rice, wheat or maize) and the mode used by the pipeline to obtain the MAGs (SASB or CACB).

Afterwards, the MAGs retrieved under each condition were used as input for MAGFlow/BIgMAG, and leveraging on these outcomes it is possible to notice how CACB allowed to recover a higher number of MAGs in the case of maize and rice, and this pipeline setting also enabled the possibility to have at least 1 MAG with mid–quality for wheat and maize ( Figure 3a, b). Nonetheless, nor the Kruskal–Wallis test ( *p*–value *=* 0.25455 considering MAGs annotated at genus level) nor the Duncan test showed significant differences among the samples using SASB or CACB if considered a threshold value of 0.05; this kind of scenario is not necessarily negative since in some cases the user could be studying samples from the same matrix throughout time in which it is desirable to account with a stable and permanent communities without differences among them.

On the other hand, taking into account the overall outcomes of this sample analysis, MAGFlow/BIgMAG underlines the advantages CACB permits by increasing both the quality and the number of MAGs obtained per type of assembly. This is supported by the higher proportion of annotated MAGs at genus levels for all soil/rhizosphere samples treated under CACB mode ( Figure 3c). These observations can even lead to cluster samples assembled under CACB in the same group when they belong to different matrices ( Figure 3d). Similar outcomes have been published by Vosloo et al. (2021) during experiments set with this type of operational mode, fixing metaSPAdes as assembler and MetaBAT2 as binning software.

Likewise, when the rice samples were assembled and binned under CACB mode, a higher number of MAGs was obtained, compared to wheat and maize samples, even though all the co-assemblies accounted with a similar number of reads (sequencing depth). This situation highlights how the sequencing depth is not the single factor affecting the assembly process and stresses the need to consider how additional conditions can influence the results such as the error rate of the sequencing method, the complexity of soil/rhizosphere samples, and the read length ( Sims et al., 2014).

**Figure 4.**
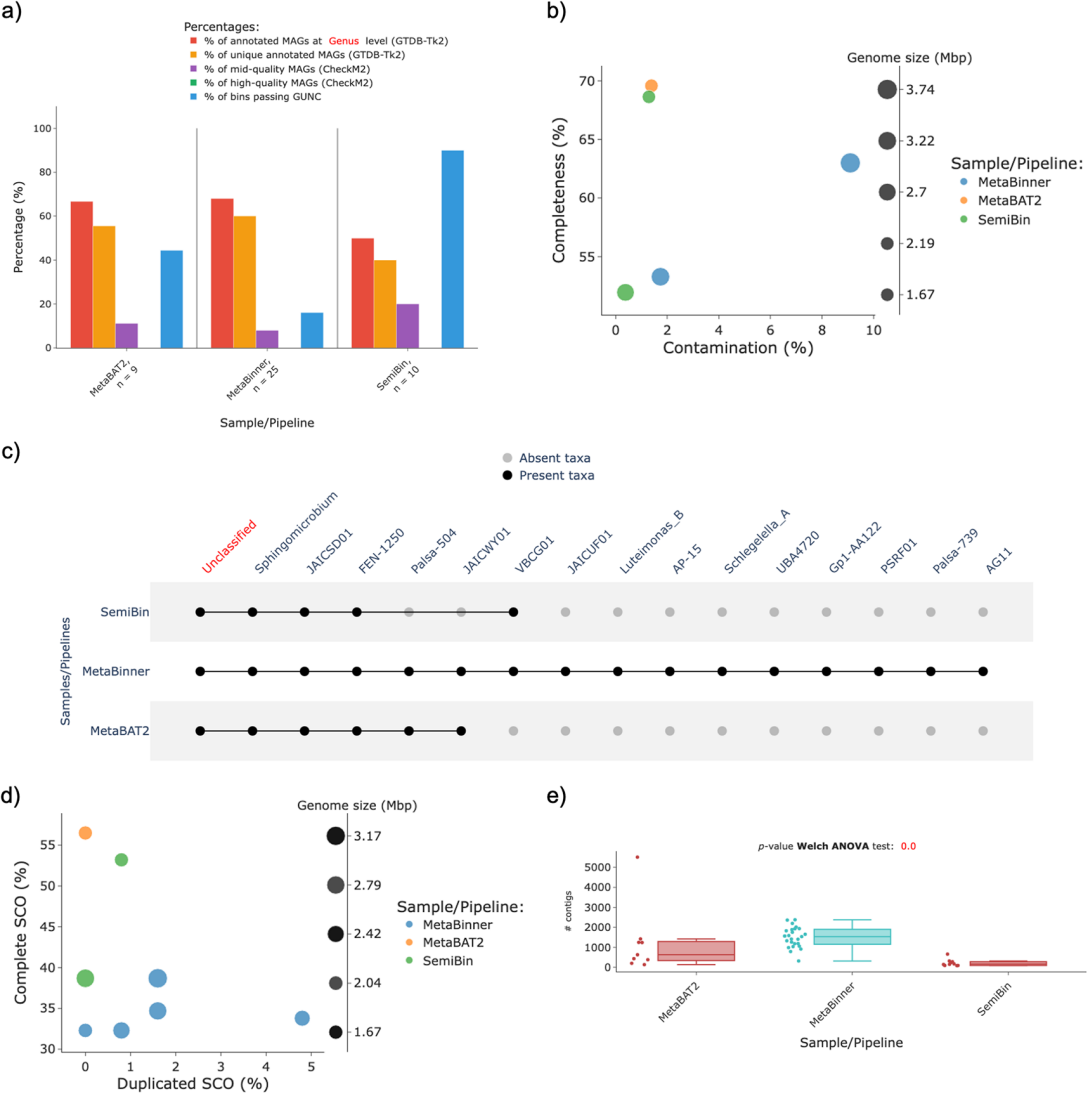
MAGFlow/BIgMAG results of the *in-silico* benchmarking of MAGs obtained through several tools using a single co-assembly of rice soil samples: *a)* bar plot summary of different features ( *see* main text) per sample, *b)* scatterplot of the completeness level against contamination portion of mid–quality MAGs, *c)* presence/absence matrix representation depicting annotated taxa in each sample at genus level, *d)* dispersion of the MAGs according to the presence of complete and duplicated SCO and *e)* distribution of the number of contigs per MAG obtained by each binner.

Further, the co-assembly of the rice soil samples (PRJNA663614) generated by nf–core/mag was used to build MAGs using MetaBinner ( Wang et al., 2023) and SemiBin ( Pan et al., 2023) in their default configurations, benchmarking them against the performance of MetaBAT2 (the version included in nf–core/mag). By these means, the MAG quality metrics of different binning tools were analyzed using MAGFlow/BIgMAG ( v1.1.0); the outcomes are presented in Figure 4. From these results, it is noticeable how using the same assembly MetaBinner recovered a higher number of MAGs, and hence reporting genera that nor SemiBin nor MetaBAT2 were able to reconstruct ( Figure 4a, c). Nonetheless, although MetaBinner produces a greater number of MAGs, the quality of these is low, given that only two of the 25 MAGs from MetaBinner falls into the mid–quality category ( Figure 4b); outcomes in the same line are depicted by the BUSCO plot, since only 5 MAGs depict at least 30% of SCO with less than 5% of these SCO duplicated ( Figure 4d).

On the other hand, the *p*–value of the Welch ANOVA for the variable *Number of contigs* per MAG depicts a significant difference among the binners, from which it is noteworthy that SemiBin groups a lower number of contigs to generate each MAG ( Figure 4e).

## Discussion

The strategy MAGFlow uses to perform the analysis attempts to follow the nf–core guidelines (P. A. Ewels et al., 2020), which allows robustness and reproducibility, and it has been tested using nf–test. This is supported by its Nextflow wrapping, along with its great performance features such as the possibility to run the software independently and in parallel, the chance to couple it to many other Nextflow modules or pipelines and the property to be scalable and adjusted to the available resources on the system user ( Di Tommaso et al., 2017). MAGFlow can also be implemented through a wide variety of profiles such as Conda, Mamba, Docker, Singularity and Apptainer, which increases the scope of suitable systems to execute it. In addition, the pipeline can be launched in local environments, HPC clusters featuring executors as SLURM or SGE, or cloud–based solutions such as Azure Batch or AWS Batch (native Nextflow functionality not tested for MAGFlow).

At the moment of writing this report, another specific workflow (GENcontams.nf) to measure the quality of MAGs through multiple tools is available, which is part of the GEN–ERA toolbox ( Cornet et al., 2022). Among the advantages of this package, we can enumerate the inclusion of tools that MAGFlow currently lacks, such as Physeter, Kraken2 and EukCC, the wide control the user can apply over the parameters to run the software, as well as the integration with the GEN–ERA suite. Nonetheless, GENcontams.nf is written under Nextflow DSL1, which is no longer supported by the developers, it does not incorporate a visualization module, and it is not modularized, meaning that all of the analysis are performed in a single script. This single-script configuration increases the difficulty to track and monitor the pipeline execution, disables the feature to resume the pipeline given the lack of checkpoints, and limits the possibility to couple it to nf–core pipelines. Besides, GENcontams.nf does not perform the taxonomical annotation with GTDB-Tk2, leaving this task to a different module within the GEN–ERA toolbox.

Another pipeline called Metaphor ( Salazar et al., 2022) can provide important and informative quality metrics and plots of the reconstructed MAGs by comparing the performance of different binning software (VAMB, MetaBAT2, CONCOT), mainly through bin scores calculated by DAS Tool ( Sieber et al., 2018). However, some limitations could be pointed out: the restriction of this analysis only to the binning software enclosed by the workflow, the requirement of running the entire pipeline from the raw reads and the generation of static image plots that are not possible to be customized or further explored.

On the other hand, in terms of visualization and portability of the plots, MultiQC ( Ewels et al., 2016) represents the most used and widespread visualization tool, commonly included in several metagenomics pipelines that has eased the process of output integration from several pieces of software; however, out of the tools implemented in MAGFlow, MultiQC only accounts with support for QUAST and BUSCO data obtained from their execution elsewhere.

MAGFlow/BIgMAG attempts to bypass these limitations in the field of metagenomics data analysis given the reproducibility and scalability of its workflow to assess the quality of the MAGs. Likewise, our tool accounts with diverse functionalities including taxonomic classification, visualization of assembly metrics and the estimation of structural integrity. Not only the advantages of MAGFlow/BIgMAG are reflected on the scientific scope, but also in practical terms since it accounts with a modularized Nextflow architecture to enclose the software tools and the easiness to analyze different types of input and several MAG folders (recommended up to 15) in a single run. Additionally, BIgMAG complements MAGFlow by generating high–quality automatic plots for all the encompassed tools, allowing a high degree of interactivity and customization according to the user needs. As a result, MAGFlow/BIgMAG represents a unique tool to our knowledge that integrates the execution of the software with a visualization module to extract MAG quality information and taxonomical annotation, featuring MAGFlow/BIgMAG as a helpful piece of software when targeting comparisons among different metagenomics pipelines or tools to bin contigs.

Furthermore, MAGFlow/BIgMAG can be useful during the execution of studies aimed to detect the presence of MAG hidden contamination after the binning step. For instance, our tool could automatize part of the work during the analysis of the effects of multi–coverage metagenomic binning ( Mattock & Watson, 2023), since some of the tools used in such study to measure the MAG quality and perform taxonomical annotation are also enclosed by MAGFlow/BIgMAG.

Also, MAGFlow/BIgMAG provides a convenient support during exploratory analyses that involve establishing general differences across samples as we showed with the examples presented in this paper.

Finally, following the principle of divide–and–rule proposed by Lupo et al. (2021), which suggests that genome quality and contamination should be assessed with as many different tools as possible, MAGFlow/BIgMAG scope will be continuously expanded through the inclusion of additional tools, such as DAS Tool, Kraken2, Physeter, EukCC, among others.

## Data Availability

The raw sequencing data to test MAGFlow/BIgMAG were retrieved from the Sequence Read Archive (SRA), and they can be accessed through the following identifiers: Sequence Read Archive: NextSeq500 of rice rhizosphere soil: Francis booting rep1. Accession number SRR12687830; https://www.ncbi.nlm.nih.gov/sra/?term=SRR12687830 ( Fernández-Baca et al., 2021). Sequence Read Archive: NextSeq500 of rice rhizosphere soil: Francis booting rep2. Accession number SRR12687829; https://www.ncbi.nlm.nih.gov/sra/?term=SRR12687829 ( Fernández-Baca et al., 2021). Sequence Read Archive: NextSeq500 of rice rhizosphere soil: Francis booting rep3. Accession number SRR12687818; https://www.ncbi.nlm.nih.gov/sra/?term=SRR12687818 ( Fernández-Baca et al., 2021). Sequence Read Archive: AAFC-Pcyc_02A_Pos1_Plot18_noN_noP_2016-08. Accession number SRR7013867; https://www.ncbi.nlm.nih.gov/sra/?term=SRR7013867 ( Li et al., 2020). Sequence Read Archive: AAFC-Pcyc_12A_Pos1_Plot73_noN_noP_2016-08. Accession number SRR7013874; https://www.ncbi.nlm.nih.gov/sra/?term=SRR7013874 ( Li et al., 2020). Sequence Read Archive: Rhizosphere soil 1. Accession number SRR12192850; https://www.ncbi.nlm.nih.gov/sra/?term=SRR12192850 ( Akinola et al., 2021). Sequence Read Archive: Rhizosphere soil 2. Accession number SRR12192849; https://www.ncbi.nlm.nih.gov/sra/?term=SRR12192849 ( Akinola et al., 2021). Sequence Read Archive: Rhizosphere soil 3. Accession number SRR12192848; https://www.ncbi.nlm.nih.gov/sra/?term=SRR12192848 ( Akinola et al., 2021). Sequence Read Archive: Metagenome_ID964_ATCC. Accession number SRR8359173; https://www.ncbi.nlm.nih.gov/sra/?term=SRR8359173 ( Portik et al., 2022). Sequence Read Archive: WGS of ATCC MSA-1003 Mock Microbial Community with PacBio CCS on the Sequel II System. Accession number SRR9328980; https://www.ncbi.nlm.nih.gov/sra/?term=SRR9328980 ( Portik et al., 2022). The bash scripts with all required configurations to run each pipeline as we performed in this paper, the MAGs recovered by each pipeline or binner using the mock community or soil/rhizosphere samples, as well as the outputs generated by MAGFlow/BIgMAG during the analysis of these MAGs have been made available in a Zenodo repository. Zenodo: Metagenome quality metrics and taxonomical annotation visualization through the integration of MAGFlow and BIgMAG (Sup. Material). https://zenodo.org/records/13628330 (v1.2). This project contains the following underlying data:
• The recovered MAGs by 6 different metagenomics pipelines (ATLAS, DATMA, MetaWRAP, MUFFIN, nf-core/mag and SnakeMAGs) using a mock community as input (SRR8359173 and SRR9328980), complemented with the output from MAGFlow (v1.0.0) using these MAGs as input for their quality assessment and taxonomical annotation. • The MAGs produced by nf-core/mag using rice/rhizosphere sequenced libraries (PRJNA663614, PRJNA448773 and PRJNA645385) in either single assembly/single binning or co-assembly/co-binning mode, complemented with the output from MAGFlow ( v1.0.0) using these MAGs as input for their quality assessment and taxonomical annotation. • Scripts, commands and configuration files to run the different pipelines (ATLAS, DATMA, MetaWRAP, MUFFIN, nf-core/mag and SnakeMAGs) and reproduce the experimental conditions. • Outputs, commands and scripts to run Metabinner and Semibin in their default configuration using the rice soil samples co-assembly, along with the MAGFlow ( v1.1.0) output to compare these binners against MetaBAT2. Data are available under the terms of the Creative Commons Attribution 4.0 International license (CC-BY 4.0).

## Software availability

The MAGFlow/BIgMAG source code is freely available as GitHub repositories, and it can be used under an MIT license. A detailed tutorial to install the dependencies, configuration setting instructions, practical visualization tips, a demo video and example commands are documented on the repositories.

MAGFlow:

Source code: https://github.com/jeffe107/MAGFlow.

Archived source code of v1.1.0: https://doi.org/10.5281/zenodo.13628774

BIgMAG:

Source code: https://github.com/jeffe107/BIgMAG.

Archived source code of v1.1.0: https://doi.org/10.5281/zenodo.13628741

License: MIT license.
