# Supplementary material for: Metagenome quality metrics and taxonomical annotation visualization through the integration of MAGFlow and BIgMAG
Source: F1000Res. 2024 Sep 23;13:640. Originally published 2024 Jun 17. [Version 2] doi: 10.12688/f1000research.152290.2 (PMC11445639; doi:10.12688/f1000research.152290.2)
Supplement: Peer review history

# Reviewer report: Reviewer response for version 2

**DOI:** 10.5256/f1000research.171258.r326143

**Published:** 2024-11-06

**Authors:** Abraham Gihawi

**Affiliations:** 1 University of East Anglia, Norwich, UK

Jeferyd Yepes-García and Laurent Falquet present the revised article.

 I appreciate the work that the authors have put into updating the software. I am glad to see that my previous debugging efforts have been implemented. Unfortunately, I am still unable to get the software to work. I also appreciate that this seems to be in contrast to the other reviewers who have now found the software to work following the revisions. I acknowledge that the authors have requested contact via GitHub issues, however the editorial team has expressed a preference for this to be kept within the review process.

 I have been trialling this software on a linux based high-performance computing cluster: CentOS Linux release 7.9.2009 (Core), without administrator access. I have been using nextflow version 24.04.2.5914 and openjdk version "20.0.2" 2023-07-18. Each job has been launched to a SLURM job scheduler requesting 350GB of RAM with ample wall time limits.

 I tried the test command as instructed by the authors:

 nextflow run MAGFlow/main.nf -profile test,singularity --outdir test_out

 I tried this with various parameters with singularity (cleared cache, version 3.6.0), conda (version 4.10.3), mamba (version 0.15.3) and podman (version 1.6.3) but was unable to get it working. With each environment it seems to be a different issue.

Are the conclusions about the tool and its performance adequately supported by the findings presented in the article?

Partly

Is the rationale for developing the new software tool clearly explained?

Partly

Is the description of the software tool technically sound?

Yes

Are sufficient details of the code, methods and analysis (if applicable) provided to allow replication of the software development and its use by others?

Yes

Is sufficient information provided to allow interpretation of the expected output datasets and any results generated using the tool?

Yes

Reviewer Expertise:

microbial bioinformatics and metagenomics

I confirm that I have read this submission and believe that I have an appropriate level of expertise to confirm that it is of an acceptable scientific standard, however I have significant reservations, as outlined above.

# Reviewer report: Reviewer response for version 2

**DOI:** 10.5256/f1000research.171258.r326145

**Published:** 2024-10-04

**Authors:** Fotis Baltoumas

**Affiliations:** 1 BSRC "Alexander Fleming, Vari, Greece

In the revised version, the authors have addressed my concerns. The MAGflow pipeline now installs and works correctly in the testing environments I have applied.

 Therefore, I find the paper suitable to be accepted.

Are the conclusions about the tool and its performance adequately supported by the findings presented in the article?

Yes

Is the rationale for developing the new software tool clearly explained?

Yes

Is the description of the software tool technically sound?

Yes

Are sufficient details of the code, methods and analysis (if applicable) provided to allow replication of the software development and its use by others?

Partly

Is sufficient information provided to allow interpretation of the expected output datasets and any results generated using the tool?

Yes

Reviewer Expertise:

Bioinformatics, metagenomics, sequence analysis

I confirm that I have read this submission and believe that I have an appropriate level of expertise to confirm that it is of an acceptable scientific standard.

# Reviewer report: Reviewer response for version 2

**DOI:** 10.5256/f1000research.171258.r326144

**Published:** 2024-10-01

**Authors:** Juliette Hayer

**Affiliations:** 1 UMR MIVEGEC (University of Montpellier- IRD-CNRS), Montpellier, France

The authors have added useful information, and answered my questions and comments. I am satisfied with the revision work that was achieved and have no further comments to make.

Are the conclusions about the tool and its performance adequately supported by the findings presented in the article?

Yes

Is the rationale for developing the new software tool clearly explained?

Yes

Is the description of the software tool technically sound?

Partly

Are sufficient details of the code, methods and analysis (if applicable) provided to allow replication of the software development and its use by others?

Yes

Is sufficient information provided to allow interpretation of the expected output datasets and any results generated using the tool?

Partly

Reviewer Expertise:

Bioinformatics, Metagenomics, Bacterial genomics, Antimicrobial Resistance

I confirm that I have read this submission and believe that I have an appropriate level of expertise to confirm that it is of an acceptable scientific standard.

# Reviewer report: Reviewer response for version 1

**DOI:** 10.5256/f1000research.167031.r300029

**Published:** 2024-07-31

**Authors:** Juliette Hayer

**Affiliations:** 1 UMR MIVEGEC (University of Montpellier- IRD-CNRS), Montpellier, France

The authors present a new and very useful workflow, MAGFlow, coupled with a visualization tool (BIgMAG) for assessing the quality of Metagenome Assembled Genomes (MAGs). These tools allow not only to assess the quality of the MAGs by combining several metrics including the completeness and the contamination rate, but also to assign the taxonomy of the MAGs using the latest GTDB-Tk2. In the manuscript, the authors also show the utility of MAGFlow and BIgMAG for comparing various workflows for MAG reconstruction, but also various strategies of assembly and binning for metagenomes.

 The workflow seems well designed and makes use of nf-core modules, which highly valuable. The way it is coded and the use of Nextflow and containers for the different processes makes the analyses performed with this workflow scalable and reproducible.

 My comments are below, organised by section.

**Introduction:**

 I think that another similar tool exists in Anvi'o, maybe the authors could add some words about it.

**Methods:**

 I think the fact that several different kind of inputs can be used for MAGFlow is great, therefore it is not clear if the user should input bins or assemblies (paragraph 3 in the Methods for MAGFlow => assembly/binning). What would happen if the user provides the assembly without binning step? Please clarify about the different inputs.

 Regarding the Methods section for BIgMAG, the sentence: "To process the outcomes from GTDB-Tk2, BIgMAG generates a presence/absence matrix representation of annotated taxa at the taxonomical level specified by the user, in a novel strategy to extract the core data without showing the complete phylogeny of the annotated MAGs." 

 I would appreciate if the authors could provide more details about the "novel strategy to extract the core data without showing the complete phylogeny".

**Results:**

 I am wondering why only MUFFIN was tested for hybrid assembly, while nf-core/mag can also do it?

 I think it would bring interesting information to also compare the binning from different binning tools in the same pipeline (ex: nf-core/mag).

 Also, have the authors considered comparing the results obtained from binning refinement methods like DAStool (now included in nf-core/mag), which can refine bins from different binning methods on the same datasets. I think it would bring even more interesting data to provide the community, using MAGFlow and BIgMAG to show it's utility. Maybe something to include for the comparison presented in Fig3?

 Fig2. I think the legend for c) and d) are inverted.

 Regarding BIgMAG and the plot displayed on Figure 2 e): I think that the presence/absence matrix would benefit from removing the black lines that are misleading (ex. DATMA has the line going over absent taxa that are in grey...). The dots are sufficient in my opinion.

 For the crop MAGs comparison with nf-core/mag (fig3), it might be useful to provide the accession numbers of the reference genomes used for mapping out the host genome reads (reproducibility).

**Minor comments on the Discussion:**

 Paragraph2 - sentence 1: a word is probably missing, the sentence is unclear, please rephrase.

 Paragraph4 - sentence 1 is too long and unclear. The authors should break in several sentences and clarify.

 A last question: would the authors consider adapting MAGFlow/BIgMAG for an integration directly with nf-core/mag for example? if the developers are up for it of course.

Are the conclusions about the tool and its performance adequately supported by the findings presented in the article?

Yes

Is the rationale for developing the new software tool clearly explained?

Yes

Is the description of the software tool technically sound?

Partly

Are sufficient details of the code, methods and analysis (if applicable) provided to allow replication of the software development and its use by others?

Yes

Is sufficient information provided to allow interpretation of the expected output datasets and any results generated using the tool?

Partly

Reviewer Expertise:

Bioinformatics, Metagenomics, Bacterial genomics, Antimicrobial Resistance

I confirm that I have read this submission and believe that I have an appropriate level of expertise to confirm that it is of an acceptable scientific standard, however I have significant reservations, as outlined above.

# Response

**Published:** 2024-09-02

**Authors:** Jeferyd Yepes

Dear Dr. Juliette Hayer,

 We appreciate the time you took to review our manuscript and tool, and we are grateful for the valuable comments and suggestions you provided us. Please find below our answers to each of them in the same order you stated your comment/suggestion:

***Response 1:***

 The authors have noticed the power and utility of anvi’o for a wide variety of analysis in the metagenomics field. Nonetheless, anvi’o does not account with workflow nor artifact to carry out the quality measurement of bins or MAGs since anvi’o current metagenomics workflow does not include binning software. Therefore, MAGFlow/BIgMAG wouldn’t be useful to process contigs artifacts from the anvi’o suite.

***Response 2:***

 The design and purpose of BIgMAG and MAGFlow is the analysis of bins or MAGs obtained after different pipelines or binning software. Eventually, files with assemblies can used as input (in essence they are the same type of file), albeit software such as CheckM2, BUSCO or GUNC are designed to analyze “ideally” one single genome at a time. As a result, and since an assembly is a mixture of multiple genomes, the calculated contaminated portion of the input files would be over-represented, and the values of completeness and contig heterogeneity would lead to erroneous conclusions based on false estimations. The text was modified to clearly state that the input for MAGFlow is genomic files of the MAGs.

***Response 3:***

 Traditionally, to visualize the output from GTDB-Tk2, the researchers use tools such as iTOL or TreeViewer in which a work of branch collapsing should be carried out to reach the desired plot that depicts the phylogeny and annotation of the input genomes. In the case of BIgMAG, the output from GTDB-Tk2 is handled in the sense of processing only the annotation of the input genomes (the *.tsv* summary) and not using the entire phylogeny, this is translated into a matrix of presence/absence at the taxonomical level selected by the user. Consequently, we consider that this strategy allows for a cleaner view of the taxonomy across samples.

***Response 4:***

 Indeed, nf-core/mag is able as well to perform hybrid assembly. In order to complement the scope of our comparison, we tested nf-core/mag performing a hybrid assembly of the mock community. Therefore, the manuscript result section was modified to include these outcomes.

***Response 5:***

 Precisely, among the advantages of MAGFlow/BIgMAG is to compare different binning tools in terms of their performance to provide more complete and less contaminated bins. To expand this utility, we have performed a comparison among MetaBAT2, SemiBin and Metabinner using the same assembly. The results from this comparison are now included in the new version of the manuscript.

***Response 6:***

 We thank the reviewer for spotting this inversion. It has been reviewed and corrected for the new version of the manuscript.

***Response 7:***

 In some cases, the line connecting the dots can be misleading when the length of the list of the taxonomical group is short. Nonetheless, when the users have a long list of taxonomical groups, *i.e.,* genus, the plot is depicted quite compacted in the default state, and the user has to use the sliding window, the line shows that there is still genus present in a specific sample.

***Response 8:***

 We acknowledge the reviewer for this suggestion, and hence the manuscript has been modified to include this accession numbers.

***Response 9:***

 We modified that sentence to provide more clarity to the reader.

***Response 10:***

 We thank the reviewer for the comment, and we modified this specific sentence to provide more clarity to the reader.

***Response 11:***

 We have considered the integration with nf-core/mag since our pipeline is Nextflow based and we attempt to follow the nf-core guidelines. In this sense, we would be interested in integrating our tool by developing a parallel application (similar to our app_lite.py script implementation) to maintain the possibility for the user to run MAGFlow/BIgMAG separately from nf-core/mag to analyze the outcomes from different pipelines or binning software, and not force them to use only nf-core/mag. As a result, we could develop an nf-core module able to process the output from nf-core/mag to generate a single dashboard html file displaying the plots and metrics calculated by the pipeline.

# Reviewer report: Reviewer response for version 1

**DOI:** 10.5256/f1000research.167031.r300036

**Published:** 2024-07-29

**Authors:** Fotis Baltoumas

**Affiliations:** 1 BSRC "Alexander Fleming, Vari, Greece

The article by Yepes-Garcia and Falquet, titled "Metagenome quality metrics and taxonomical annotation visualization through the integration of MAGFlow and BIgMAG" presents a new, nextflow-based pipeline for the analysis and assessment of MAGs.

 Although a number of similar workflows exist, I believe MAGflow/BIgMAG to be a welcome addition, especially since their combination seems to offer a number of visualization and analysis schemes not available by other solutions.

 The manuscript is well-written and in good English. The methodology, case studies, and discussion are presented in adequate detail, including statistical analysis and interpretation. Overall, the presentation of the workflow is sound.

 However, I do have one major point that needs to be addressed, before I can consider the manuscript to be 100% acceptable for MEDLINE indexing:

 I was not able to initiate and run the MAGflow workflow, even the test runs. The reason for this is that at least some of the dependencies of the workflow either don't exist anymore, at least publicly or their name/version/installation method has been updated. For example, this happens with the "biocontainers/checkm2" dependency.  I have tried to test MAGflow using conda, mamba AND docker, and in ALL 3 cases, the workflow fails saying that the aforementioned package either doesn't exist or it is not publicly available.

 (specifically, docker gives the following error report:

 Command error:

   Unable to find image 'biocontainers/checkm2:1.0.1--pyh7cba7a3_0' 

   docker: Error response from daemon: pull access denied for biocontainers/checkm2, repository does not exist or may require 'docker login': denied: requested access to the resource is denied.

 )

 I have tried to run this in 3 different machines, all of which have run other nextflow pipelines successfully, and all of which have conda/mamba and docker installations that work as intended.

 The authors need to update the dependencies and setup scripts of their pipeline, and make sure that:

 1. it depends solely on publicly available dependencies. If something is proprietary or requires access to be provided by the authors of the package, it should be stated explicitly. 

 Can be run successfully, without any troubleshooting required by the end user.

Are the conclusions about the tool and its performance adequately supported by the findings presented in the article?

Yes

Is the rationale for developing the new software tool clearly explained?

Yes

Is the description of the software tool technically sound?

Yes

Are sufficient details of the code, methods and analysis (if applicable) provided to allow replication of the software development and its use by others?

Partly

Is sufficient information provided to allow interpretation of the expected output datasets and any results generated using the tool?

Yes

Reviewer Expertise:

Bioinformatics, metagenomics, sequence analysis

I confirm that I have read this submission and believe that I have an appropriate level of expertise to confirm that it is of an acceptable scientific standard, however I have significant reservations, as outlined above.

# Response

**Published:** 2024-09-02

**Authors:** Jeferyd Yepes

Dear Dr. Fotis Baltoumas,

 We appreciate the time you took to review our manuscript and tool, and we are grateful for the valuable comments and suggestions you provided us.

 We are sorry that you didn’t manage to run MAGFlow. For this reason, we carried out many more tests of MAGFlow under the following settings:

- Rocky Linux version 8.10 with profiles Apptainer and Mamba.
- WSL running with Ubuntu 22.04.4 with profiles Singularity, Docker, and Mamba.

Within these environments the pipeline worked appropriately using the recommended versions of Java and Nextflow in the manuscript. Also, the versions of the profile software in which MAGFlow was tested is now indicated in the new version of the manuscript.

 Finally, we kindly invite you to review our paper after applying the discussed changes. In addition, in case of persisting inconveniences with the tool, please open an issue on the github repository.

# Reviewer report: Reviewer response for version 1

**DOI:** 10.5256/f1000research.167031.r300031

**Published:** 2024-07-18

**Authors:** Abraham Gihawi

**Affiliations:** 1 University of East Anglia, Norwich, UK

Jeferyd Yepes-García and Laurent Falquet present the article entitled: "Metagenome quality metrics and taxonomical annotation visualization through the integration of MAGFlow and BIgMAG". This article describes two pieces of software. Firstly MAGFlow, which is concerned with the quality control and taxonomy of contigs. Secondly BIgMAG presents a solution to visualising these results.

 Overall the pipeline is similar to others available, but could still offer considerable use to the community if it is a well written and a well maintained piece of software. Care needs to be taken in the manuscript, particularly with results that points are backed up by figures and statistical tests.

 Despite investing a significant amount of time trying, I have not been able to get the pipeline to run. Every time it seems to be a different error arising.

 The MAGflow software doesn't seem to work with my own gtdb-tk v2 database. The tool seems to want to re-download it despite giving it a valid absolute file path, it raised an error in downloading the database. I updated line 6 ((gtdbtk2_download_db.sh) to "wget https://data.ace.uq.edu.au/public/gtdb/data/releases/latest/auxillary_files/gtdbtk_package/full_package/gtdbtk_data.tar.gz". Despite this, I still couldn't get the GTDB-tk step to run. Separately, there seem to be a range of other bugs that keep cropping up and I invested a significant amount of time to try and resolve each one. Having been unable to resolve all of them I am raising it in this review that I recommend that the authors carry out some additional user testing of the software. I am using the singularity profile on a linux based computing environment and launching it with:

 "nextflow run MAGFlow/main.nf -profile singularity --files 'samples/*' --outdir /path/to/magflow_test/mag_output -qs 1"

 Resulting Error:

 "

 ERROR ~ Error executing process > 'MAGFlow:BUSCO (XXXXXXXX)'

 Caused by:

   Process `MAGFlow:BUSCO (XXXXXXXX)` terminated with an error exit status (1)

 Command executed:

   busco -i XXXXXXXX         -o busco         -m genome -c 8 --force         -l bacteria_odb10

   cat <<-END_VERSIONS > versions.yml

   "MAGFlow:BUSCO":

           busco: $( busco --version 2>&1 | sed 's/^BUSCO //' )

   END_VERSIONS

 Command exit status:

   1

 Command output:

   this version of pandas is incompatible with numpy < 1.17.3

   your numpy version is 1.16.4.

   Please upgrade numpy to >= 1.17.3 to use this pandas version

   There was a problem installing BUSCO or importing one of its dependencies. See the user guide and the GitLab issue board (https://gitlab.com/ez$

 Command error:

   this version of pandas is incompatible with numpy < 1.17.3

   your numpy version is 1.16.4.

   Please upgrade numpy to >= 1.17.3 to use this pandas version

   There was a problem installing BUSCO or importing one of its dependencies. See the user guide and the GitLab issue board (https://gitlab.com/ez$

 "

 As I could not carry out the first step, I was unable to test BIgMAG. I have also included some suggestions for the manuscript below.

 Suggestions:

 - "high-quality MAGs depict levels of contamination below 5% and completeness above 90%" - I feel it would be appropriate to cite Bowers et al. here that established the MIMAG standards - https://doi.org/10.1038/nbt.3893 

 - "Nevertheless, many pipelines or methodologies only include one or two tools to measure the quality of the MAGs" - then cite Krakau et al. 2022 [Ref-1]- This tool has several evaluation tools including the ones that this proposed pipeline offers - GTDB-tk, BUSCO, CheckM, GUNC, QUAST. This is more than 'one or two tools' to measure MAG quality. Similarly, Tadrent et al. details CheckM, GUNC, GTDB-Tk and CoverM which is also more than 'one or two tools'. The text would benefit from expanding upon these tools and state clearly how the work of the authors distinguishes from these. Is it purely the additional visualisation that the authors provide?

 - It has been shown that single sample metagenome assembly can cause extensive hidden contamination. Mattock J, et al., 2023 [Ref-2] - How does the pipeline proposed by the authors handle or investigate this?

 - "Following the GTDB-Tk2 plot, as an attempt to compare the features among samples and cluster them according to their similarity using different parameters provided by BUSCO, CheckM2, GUNC and/or GTDB-Tk2, a cluster heatmap is displayed. To render this plot, average values are used, combined with the proportion of MAGs passing the GUNC test and/or the proportion of annotated MAGs by GTDB-Tk2" - Perhaps it's just me. I find this paragraph unclear at describing what exactly is being compared. Are you comparing the completeness/quality for the same features? Does this cluster the samples, or the features?

 Overall, the results need values and statistical tests to support the claims that the authors are making. A few examples are in the points below.

 - "For instance, MetaWRAP and ATLAS show a higher dispersion in terms of the number of contigs assigned per bin (Figure 2a)" - This sentence indicates that MetaWRAP and Atlas demonstrate a higher variation in terms of the number of contigs compared to all other tools. Looking at the plot, this might not be entirely true. SnakeMAGS and nf_core_mag also seem similar. This statement should be backed up with values and statistical tests.

 - Similarly the following sentence should be backed up by values and statistics: "whilst nf core/mag depicted a higher proportion of MAGs with higher quality (completeness > 70% and contamination < 5%) (Figure 2b).". Currently, this can not be interpreted from the plot. This should also be considered for sentence afterwards: "Likewise, nf-core/mag and MetaWRAP exhibit a trend to produce moreMAGswith duplicated SCO with at least 50% of complete SCO".

 - "where MetaWRAP, ATLAS, nf – core/mag and SnakeMAGs show the highest consistency and correspondence between the MAGs passing the GUNC test and the CSS value (Figure 2d)." - What do the authors mean by this? Surely it would be preferably to compare the number of results passing/failing the GUNC filter? This graph currently could suggest that more contigs are failing chimerism checks than passing?

 - "increasing the likely to fail" - Revise grammar. Additionally I don't necessarily know if something is more likely to fail because it's written in a single script. Can the authors expand on this point or back it up?

 - "MultiQC is the top of the notch application" - The grammar here needs revising and also it reads as quite informal for a manuscript. What do the authors mean by 'top of the notch'? highly cited? highly used? There is no context to this statement.

 - "making it interestingly useful when" - revise grammar

 - Methods: Thank you to the authors for uploading code on Zenodo repositories. As a minor suggestion, it might be nice for reproducibility purposes if the authors state what version of each tool they used in their analysis.

Are the conclusions about the tool and its performance adequately supported by the findings presented in the article?

Partly

Is the rationale for developing the new software tool clearly explained?

Partly

Is the description of the software tool technically sound?

Yes

Are sufficient details of the code, methods and analysis (if applicable) provided to allow replication of the software development and its use by others?

Yes

Is sufficient information provided to allow interpretation of the expected output datasets and any results generated using the tool?

Yes

Reviewer Expertise:

microbial bioinformatics and metagenomics

I confirm that I have read this submission and believe that I have an appropriate level of expertise to confirm that it is of an acceptable scientific standard, however I have significant reservations, as outlined above.

## References

[1] : nf-core/mag: a best-practice pipeline for metagenome hybrid assembly and binning. *NAR Genom Bioinform* .2022;4(1) : 10.1093/nargab/lqac007 lqac007 10.1093/nargab/lqac007 35118380 PMC8808542

[2] : A comparison of single-coverage and multi-coverage metagenomic binning reveals extensive hidden contamination. *Nat Methods* .2023;20(8) : 10.1038/s41592-023-01934-8 1170-1173 10.1038/s41592-023-01934-8 37386187

# Response

**Published:** 2024-09-02

**Authors:** Jeferyd Yepes

Dear Dr. Abrahm Gihawi,

 We appreciate the time you took to review our manuscript and tool, and we are grateful for the valuable comments and suggestions you provided us. Please find below our answers to each of them in the same order you stated your comment/suggestion:

***Response 1:***

 Dear reviewer, the link from which the database is downloaded was broken; it was corrected, and it is working properly now. We apology for the inconvenience. In addition, we would like to share with you a small but important detail when providing different versions of GTDB to MAGFlow using the parameter --gtdbtk2_db:

- Notice that when you *untar* any GTDB dabatase, it is stored under the name release*; please keep the word *release* in the name to guarantee a proper detection by the pipeline.

This and other important tips are given in the README file of the pipeline ( https://github.com/jeffe107/MAGFlow).

***Response 2:***

 Dear reviewer, to ensure the functionality of the pipeline, we carried out several tests of MAGFlow under the following settings:

- Rocky Linux version 8.10 with profiles Apptainer and Mamba.
- WSL running with Ubuntu 22.04.4 with profiles Singularity, Docker and Mamba.

Within these environments the pipeline worked appropriately using the recommended versions of Java and Nextflow in the manuscript. Also, the versions of the profile software in which MAGFlow was tested is now indicated in the new version of the manuscript.

***Response 3:***

 The error related to pandas and Numpy incompatibility is quite rare since the pipeline runs under either containerized versions of the software or Conda/Mamba environments to ensure precisely that this kind of errors does not happen. From what we could tell, it may be possible that in your system there is an available version of BUSCO and hence the pipeline is crashing by trying to use that version which is incompatible. As we stated previously, we tested in different environments and profiles, and the pipeline worked properly.

***Response 4:***

 We appreciate your suggestion, and we included this reference in the new version of the manuscript.

***Response 5:***

 Indeed, the mentioned pipelines have evolved throughout time to include more tools and not only one or two. This statement has been changed in the manuscript the demonstrate that the power of our tool relies in the practical integration with a dashboard and the possibility it offers to compare the output from different pipelines or binning software. The mentioned pipelines, nf-core/mag, ATLAS and others, include these tools to measure the quality of the obtained bins, but they are available only if running the entire workflow. We have highlighted this advantage of our approach in the text as well.

***Response 6:***

 The challenge researchers face when building bins from assemblies of metagenome samples has been pointed out widely, and in general terms, it has been advised to carry out a co-assembly/co-binning of the samples. Our tool MAGFlow/BIgMAG can help during the investigation of MAG hidden contamination by offering quality metrics and comparisons among tools run under different binning settings. For instance, we carried out a comparison using MAGFlow/BIgMAG among two different settings to reconstruct the MAGs: single-assembly/single-binning and co-assembly/co-binning for soil-associated samples. This utility of MAGFlow/BIgMAG has been highlighted in the discussion of the manuscript.

***Response 7:***

 We thank the reviewer for this comment. This paragraph has been modified to clarify the purpose of the plot. The cluster heatmap attempts to show how similar or dissimilar the samples are using as many features as possible from CheckM2, BUSCO, GUNC and if performed, GTDB-Tk2.

***Response 8:***

 Dear reviewer, to backup this and other statements in regard to the outcomes of our experiments, we have added the result of a Welch ANOVA to the plot that shows the QUAST metrics distribution. This test helps to detect if there are significant variations in at least one of the samples, and it was chosen because it might be possible that the user does not account with the same sample size for all of their samples. This aspect has been also included and discussed in the new version of the manuscript.

***Response 9:***

 We acknowledge the reviewer for this comment, and we have reformulated our analysis of the benchmarking among pipelines by modifying this and other statements according to the new version of BIgMAG, in which we carried out a statistical analysis using features such as Number of bins, Number of unique annotated bins, Number of mid-quality MAGs, Number of high-quality MAGs and the Number of bins passing GUNC. As a result, the dashboard now accounts with a Summary section to display the results of a Kruskal-Wallis test and a Duncan test. In addition, BIgMAG is mainly thought to be an exploratory analysis tool that can assist the researchers in identifying important aspects to consider from their samples, highlighting features that can be further studied.

***Response 10:***

 The GUNC plot attempts to show the distribution of the selected parameter for each sample, discriminated by bins that passed or failed the GUNC filter. In the case of the Clade Separation Score (CSS), it can be noticed that in general terms for the bins that did not pass the filter, the CSS presented a higher dispersion and non-uniform distributions compared to the bins that passed the filter.

 The percentage of bins passing/failing the test can be visualized in the new plot included at the Summary dashboard.

 This graph does not suggest that more contigs are failing/passing the test, it only tries to show the distribution of the CSS parameter according to the bins that failed/passed the test.

***Response 11:***

 We acknowledge the reviewer for this suggestion, and therefore the phrase was modified to: “This single-script configuration increases the difficulty to track and monitor the pipeline execution, disables the feature to resume the pipeline given the lack of checkpoints, and limits the possibility to couple it to nf–core pipelines”.

***Response 12:***

 The phrase was modified to: “MultiQC represents the most used and widespread visualization tool, commonly included many metagenomics pipelines”.

***Response 13:***

 The phrase was modified to: “…featuring MAGFlow/BIgMAG as a helpful piece of software when…”

***Response 14:***

 The authors have modified the manuscript to depict the version used for the analysis in the manuscript.

 Finally, we kindly invite you to review our paper after applying the discussed changes. In addition, in case of persisting inconveniences with the tool, please open an issue on the github repository.
